# Low genetic diversity and recent demographic expansion in the red starfish *Echinaster sepositus* (Retzius 1816)

**DOI:** 10.1038/srep33269

**Published:** 2016-09-15

**Authors:** Alex Garcia-Cisneros, Creu Palacín, Yousra Ben Khadra, Rocío Pérez-Portela

**Affiliations:** 1 Animal Biology Department and Biodiversity Research Institute (IRBIO), Barcelona University, Avda. Diagonal, 643, Barcelona, Spain; 2 Center of Advanced Studies of Blanes (CSIC-CEAB), Accès cala St. Francesc, 14, Blanes, Spain; 3 Laboratoire de Recherche Génétique, Biodiversité et Valorisation des Bioressources, Institut Supérieur de Biotechnologie de Monastir, Av. Tahar Haddad, 5000, Monastir, Tunisia

## Abstract

Understanding the phylogeography and genetic structure of populations and the processes responsible of patterns therein is crucial for evaluating the vulnerability of marine species and developing management strategies. In this study, we explore how past climatic events and ongoing oceanographic and demographic processes have shaped the genetic structure and diversity of the Atlanto-Mediterranean red starfish *Echinaster sepositus*. The species is relatively abundant in some areas of the Mediterranean Sea, but some populations have dramatically decreased over recent years due to direct extraction for ornamental aquariums and souvenir industries. Analyses across most of the distribution range of the species based on the mitochondrial cytochrome *c* oxidase subunit I gene and eight microsatellite loci revealed very low intraspecific genetic diversity. The species showed a weak genetic structure within marine basins despite the *a priori* low dispersal potential of its lecithotrophic larva. Our results also revealed a very recent demographic expansion across the distribution range of the species. The genetic data presented here indicate that the species might be highly vulnerable, due to its low intraspecific genetic diversity.

In marine benthic species with limited mobility during adulthood, larval dispersal plays an important role in the intraspecific distribution of genetic diversity, and in both the genetic structure and connectivity of populations^1^. Traditionally, planktotrophic larvae have been considered to have higher dispersal capability than lecithotrophic larvae^2^,^3^,^4^,^5^,^6^. Hence, species with lecithotrophic larvae that exhibit philopatric behaviour are expected to show more genetically structured populations at finer scales^5^,^6^,^7^,^8^,^9^,^10^. Nevertheless, during recent years, several studies have demonstrated that pelagic larval duration does not directly determine the genetic structure of populations^11^,^12^. Coastal water circulation, availability of substrates, population size, fecundity and stochasticity of recruitment success may determine the different level of genetic structure found in many nearshore benthic species^13^,^14^,^15^,^16^. Additionally, other factors such as major oceanographic circulation as well as geographical straits and oceanic fronts are known to act as physical barriers that prevent propagule interchanges thereby limiting connectivity between nearby areas^17^,^18^,^19^.

Along the Atlanto-Mediterranean arch, the Almeria-Oran Front is considered the real boundary between the Mediterranean Sea and the Atlantic Ocean, acting as an important barrier to gene flow in a number of marine species^20^,^21^,^22^,^23^. The real influence of this marine transition from the genetic point of view still remains controversial due to its different effects and permeability to species displaying contrasting biological features^22^,^24^,^25^,^26^. The Mediterranean Sea itself possesses a complex oceanographic circulation system^27^, divided into two sub-basins separated by the Siculo-Tunisian Strait^20^. This sea has suffered an intricate past history. The desiccation of the Mediterranean Sea, which reduced it to a series of hypersaline lakes during the so-called Messinian salinity crisis at the Mio-Pliocene transition (6–5.5 Mya) was followed by the refilling of the basin with Atlantic water^28^,^29^. More recently, the Quaternary climatic fluctuations that shaped coastal fauna of northern Europe also had a huge impact on marine fauna of southerner Europe, including that of the Mediterranean Sea. During the cyclical glacial periods, when most of the north of Europe was covered by ice sheets, the Mediterranean Sea and the southern European coasts acted as separate marine refuges^30^. These historical events have determined the evolution of coastal species across the Atlanto-Mediterranean area^20^,^31^,^32^,^33^. The complexity of the historical, palaeo-geographical and ecological processes that have occurred in the Mediterranean explains the high biodiversity and rate of endemism in this small basin^34^. While the Mediterranean Sea is considered a hotspot of marine biodiversity, it is also one of the world’s most impacted seas^35^. It is exposed to considerable anthropogenic pressures from both short-term and long-term perturbations^36^. Mitigating further impact is hence a priority and to do this we need to understand the vulnerability of Mediterranean organisms. Molecular studies of the intraspecific distribution of genetic diversity can contribute to effective management and conservation strategies. Phylogeographic information and population genetic analysis allow exploring the most important evolutionary and contemporary factors that have shaped the extant biodiversity and its geographical distribution. Therefore, molecular analysis provides data not only on inter- and intra-genetic diversities and connectivity among populations, but also on the key processes underlying the origin and maintenance of this diversity, which should be preserved whenever possible^37^.

In this paper, we analyse one of the most emblematic echinoderms found in the Mediterranean Sea, and the first starfish mentioned in Science, by Aristotle 2,300 years ago in *Historia Animalium*: the red starfish *Echinaster sepositus* (Retzius 1783). The species is distributed across the Mediterranean Sea and the temperate waters of the eastern Atlantic, from the south-eastern limit of the English Channel to Cape Verde^38^. It inhabits from shallow (from some 2 m) to deep waters, down as deep as 250 m, on sandy bottoms, rocky substrates, and within seagrass systems^39^, showing affinity for coralline algae communities^40^. Although the species can be relatively abundant in some particular areas of the Mediterranean coast, during the last decade, some populations of *E. sepositus* in the north-western Mediterranean have dramatically decreased^40^, at least partly as a result of direct extraction from nature by the ornamental aquariums and the souvenir industries. This activity is expanding rapidly in the absence of proper systems of regulation and the control of its capture^41^.

It has been reported that *E. sepositus* releases lecithotropic larvae that remain as free swimmers for no longer than 5–6 days^42^,^43^. Nevertheless, many features of the nature and behaviour of its larvae remain unclear. Whereas some authors^42^ have reported floating eggs and larvae for this species, more recent laboratory experiments have shown that both the eggs and larvae sink to the bottom of tanks after spawning (Villamor, personal communication). In accordance with its short larval development, we may expect limited dispersal potential, low connectivity between distant areas and strong genetic structure of populations; as observed for other asteroids with similar biology^5^,^44^,^45^. If this is indeed the case, these general biological characteristics would limit the potential for recovery of extinct populations via recruitment from other geographical sources. Nevertheless, in the light of the most recent studies^14^, information regarding the connectivity of a particular species should not be directly inferred from species displaying similar biological traits, since a number of biotic and abiotic factors are related to effective larval dispersal and intra-species connectivity.

Very limited published genetic information is available for *E. sepositus* from which to infer the effective dispersal and levels of gene flow. In comparison, genetic patterns of other starfish species have been widely studied in recent years^46^,^47^,^48^,^49^,^50^,^51^. The only genetic study with this species, based on one mitochondrial gene, included only a few populations from the coast of Tunisia^45^. Hence, the aim of this study is to obtain a more complete picture of the population genetic structure of *E. sepositus*, at both the evolutionary and geographical scales. We focus on the potential effect of major marine corridors, the Strait of Gibraltar and the Siculo-Tunisian Strait, on the genetic structure and connectivity; and also on the relevance of geographical distance for the divergence of populations in a species with a short-lived larval stage. This represents a twofold contribution to marine biology: a) it increases the knowledge of population genetics and phylogeography of benthic invertebrates across the Atlanto-Mediterranean transition, and between the two Mediterranean sub-basins, which is currently patchy and controversial; and b) it generates the knowledge required to understand the potential vulnerability of this species to exploitation, according to its genetic features. It is well known that the intraspecific genetic diversity of species plays a crucial role in the long-term survival of populations because it is the raw material on which natural selection acts^52^. Populations affected by human activities that result in habitat loss, pollution and overexploitation may experience a reduction in size. In turn, this may be translated to a reduction of genetic diversity due to the effects of bottlenecks, strong genetic drift and inbreeding depression, all of which can jeopardise population persistence^53^. These effects are particularly negative in species with low dispersal potential and low connectivity, because they limit the potential for recolonisation and gene interchanges with other sources.

To address our objectives, we used both mitochondrial and nuclear markers to understand past and present events shaping the geographical distribution of the intraspecific genetic diversity in *E. sepositus*. As a mitochondrial marker we chose the cytochrome c oxidase subunit I (COI) gene because of its high resolution in phylogeographic analysis^17^,^32^,^54^ and potential comparison with a number of studies in echinoderms^22^,^24^,^26^,^45^,^55^,^56^.

## Results

### Genetic diversity

**Figure 1:**
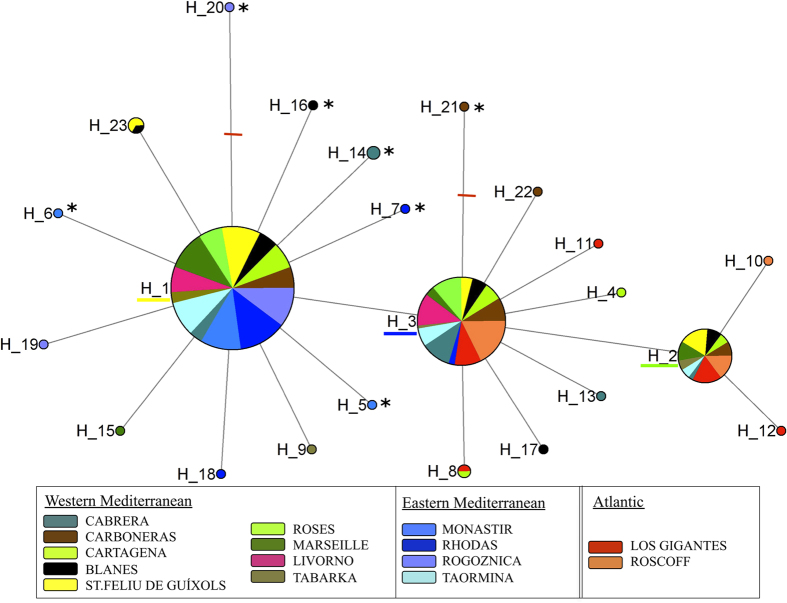
Haplotype network from COI sequences of *E. sepositus*. The area of the circles is proportional to the number of sampled individuals, and different colours represent different populations. Small red dashes represent mutation steps of more than one mutation. Asterisks (*) indicate non-synonymous mutations. The most common haplotypes, H_1, H_2 and H_3, are underlined using the colour pattern from Fig. 2.

From a total of 325 sequences (657 bp) of the COI fragment, we obtained 23 different haplotypes. Most individuals (92%) had one of three main haplotypes (H_1, H_2 and H_3), with only one mutation step between each other (see Fig. 1). We detected 18 private haplotypes, and only two of them were separated by more than one mutation step from the three most common haplotypes. Nucleotide diversity (π) and haplotype richness (Hr) for the whole dataset were 0.0013 (±1.03 . 10^−6^ SD) and 0.619 (±0.021 SD), respectively. Values of genetic diversity for COI were similar for the Atlantic and the Mediterranean basins, (Atlantic: Hr = 0.545, π = 0.00099; Mediterranean: Hr = 0.560, π = 0.00114), although within the Mediterranean basin, the eastern Mediterranean seemed to have lower values of diversity (Hr = 0.300, π = 0.00057). The population from Livorno, located in the western Mediterranean, presented only two haplotypes, and was thus the population with the fewest haplotypes and the lowest Hr (0.522).

**Figure 2:**
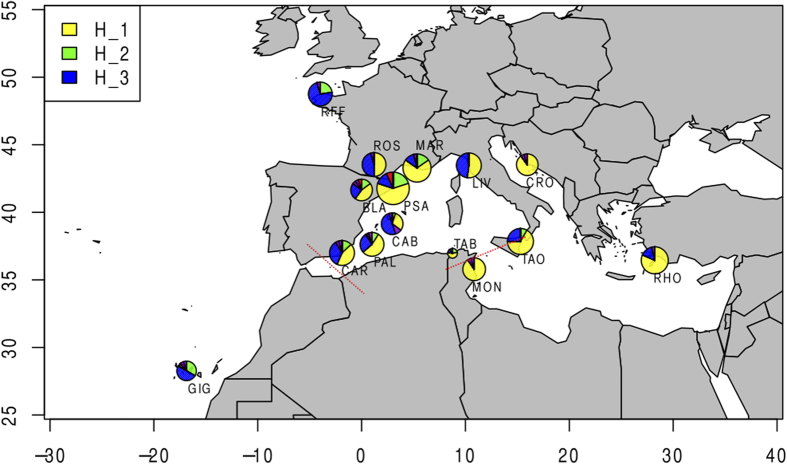
Map of the sampling locations in *E. sepositus*. Pie charts represent COI haplotype frequencies for each location, and size is proportional to the number of samples analysed. Private haplotypes are represented in purple. For location codes see Table 1. Red dotted lines represent the main marine barriers in the geographical area: the Almeria Oran Front (AOF) and the Siculo-Tunisian Strait. The map was drawn using Maps and Mapplots R packages and Inkscape v 0.48 (Free Software Foundation, Inc.; Boston, USA; http://inkscape.org).

The haplotype network obtained from COI (Fig. 1) showed a very well-connected network, separated by only a few mutation steps, and with no loops. However, 7 non-synonymous mutations were found in haplotypes that appeared infrequently. The three most frequent haplotypes appeared in most populations but displayed differences in geographical distribution. H_1, was restricted to the Mediterranean basin, whereas H_3 was not found in the eastern Mediterranean populations, with the exception of Taormina (Fig. 2).

The eight microsatellite loci showed between 6 and 32 different alleles per locus in the populations analysed. We observed differences in allelic richness among populations, but the population from Livorno displayed much lower values of allelic richness than any other population (Livorno R_s_: 2.994; all the other populations R_s_: from 3.882 to 4.340); in agreement with the occurrence of only two mitochondrial haplotypes in this population (see Table 1). There was in general a significant deficiency of observed heterozygosity (H_o_) in most populations, and populations from north-western Mediterranean and the Atlantic showed significant F_IS_ values, and Hardy-Weinberg disequilibrium (Table 1; F_IS_ values).

### Population structure and demography

**Figure 3:**
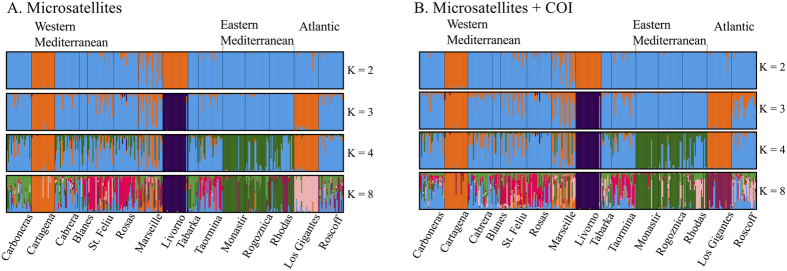
Bar plots of the Bayesian clustering analysis obtained using STRUCTURE for different K values. (**A**) Analysis based on microsatellite loci. (**B**) Analysis based on the combination of both mitochondrial sequences (COI) and microsatellite loci. Note that the same K values are presented, for comparison between databases.

The Bayesian clustering analysis performed with STRUCTURE did not revealed large differences between using only microsatellite loci or the combined microsatellite loci and COI dataset (see Fig. 3). The resolution of the analysis was better when locations were implemented as priors, under the non-admixture model (Supplementary Figures S1 and S2). The most likely K values obtained from the microsatellite loci database was used to represent the results (Fig. 3). For K = 2, only the Mediterranean populations from Cartagena and Livorno appeared separate from all the other populations. When increasing K, the Atlantic population from the Canary Islands (Los Gigantes) and all the eastern Mediterranean populations grouped into different clusters (K = 3 and K = 4). For K = 8, the main clusters detected for K = 4 were maintained (Cartagena, Livorno, Los Gigantes, and eastern Mediterranean), and all the other Mediterranean populations and Roscoff from the Atlantic appeared as a mixture of different clusters (See Fig. 3A,B and Supplementary Figure S2).

The results obtained from the analysis of molecular variance (AMOVA) for both COI and microsatellites supported the patterns obtained from STRUCTURE. The analysis revealed that most of the genetic diversity was retained, both within populations and individuals (COI: >55%; microsatellites within individuals >80%). Significant differences were observed between groups (Atlantic, and both eastern and western Mediterranean), and among populations within geographical groups for microsatellites, but not for COI. Taormina, a population on the limit between eastern and western Mediterranean, seemed to be more closely related to the western Mediterranean group. This was observed through an increase in the variation between groups for the two sets (Table 2). The other populations on the limit of the Siculo-Tunisian Strait, Tabarka and Monastir, did not show more affinity for any one specific area when moving between different sub-basins.

**Figure 4:**
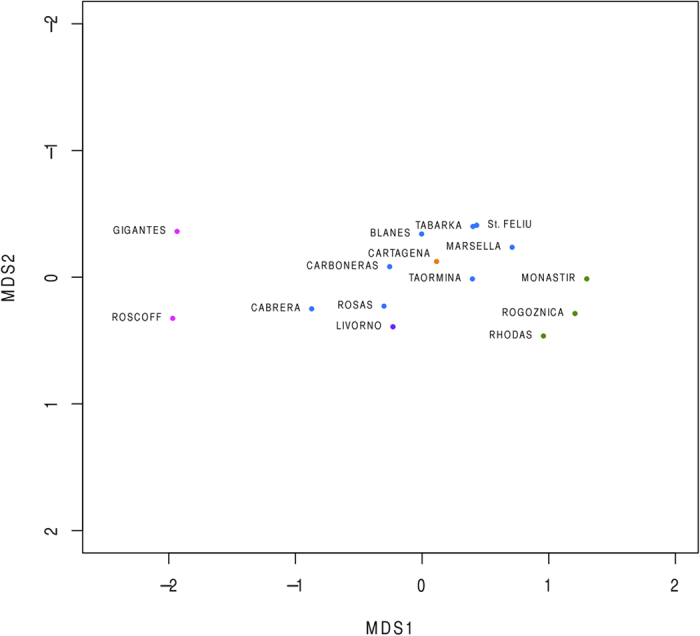
Multidimensional scaling (MDS) from the Jost’s D pairwise matrix computed from COI sequences.

**Figure 5:**
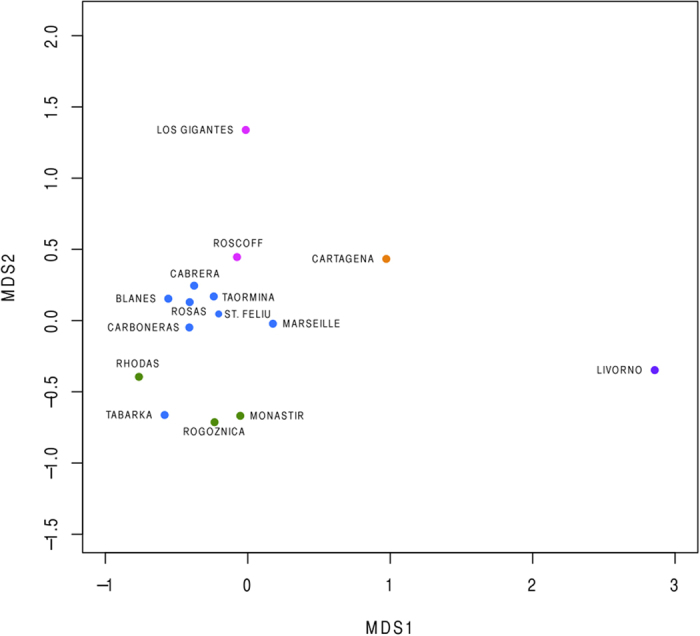
Multidimensional scaling (MDS) from pairwise Jost’s D differences from microsatellite data.

Both Φ_ST_-F_ST_ and Jost’s D values revealed significant genetic differences between populations from the Atlantic and Mediterranean basins, with only a few exceptions (Figs 4 and 5; Supplementary Tables S1 and S2). Most of the western Mediterranean populations did not show significant differences. We detected a significant positive correlation between Φ_ST_-F_ST_ and Jost’s D values (COI: r = 0.94, p = 0.001, and microsatellites: r = 0.93, p = 0.001, Supplementary Figure S3) indicating that both types of statistics detected similar genetic structure. Nevertheless, there was no significant correlation between pairwise differences between populations calculated from COI and microsatellites (correlation between Φ_ST_ and F_ST_: r = 0.10 and between Jost’s D: r = 0.13, Supplementary Figure S4). This is probably because the two types of genetic markers detect different signals of divergence between populations. In the case of the western Mediterranean populations, no significant differences in genetic structure were detected from COI; but most populations were significantly different according to the microsatellite data, which also revealed that Livorno and Cartagena were significantly different from all the other populations. With multidimensional scaling (MDS) analysis based on Φ_ST_ from COI, we observed a distribution of the populations related to geographical location, with all the eastern Mediterranean locations on the right, the western Mediterranean in the center and the two Atlantic locations on the left. Nevertheless this ordering was not maintained for the MDS based on F_ST_ from microsatellites, in which the Mediterranean populations of Livorno and Cartagena, and the Atlantic location of Los Gigantes, appeared clearly separated from all the other locations.

Genetic differentiation between populations correlated with geographic distances when the whole dataset, including both the Atlantic and Mediterranean basins, was considered together (Table 3). However, the stratified isolation by distance (IBD) analysis did not reveal IBD within the basins, indicating that the significant results were driven by regional divergence between basins^57^.

**Figure 6:**
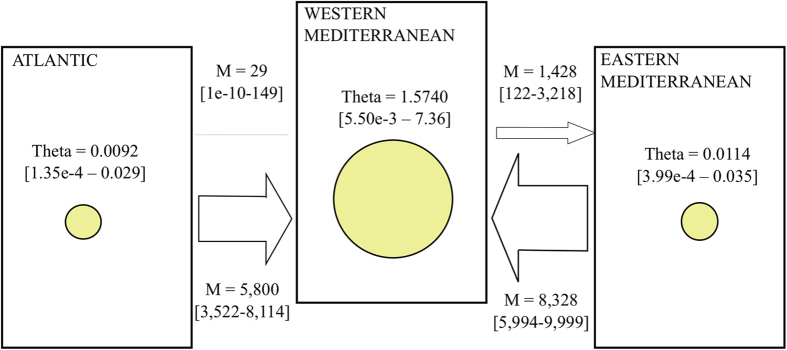
Values of *theta* and migration between basins and sub-basins. Yellow circles represent the value of *theta* (logarithmically transformed) per basin, and arrows represent the direction of migration. The thickness of the arrows is proportional to the migration value. Values in brackets are the 95% HPD confidence of *theta*.

The coalescence analysis using LAMARC based on COI sequences showed the largest effective population in the western Mediterranean sub-basin, considered as *theta* (θ) mean values, followed by the eastern Mediterranean and the Atlantic basin (Fig. 6). The estimation of effective population size showed between 33,000 and 52,000 females in the western Mediterranean sub-basin; while fewer than 500 were estimated in the other two areas (Table 4). Demographic exponential growth was detected in the eastern Mediterranean and Atlantic populations (Supplementary Figure S5). However, the growth parameter in the western Mediterranean area did not reach convergence for any of the replicates (Table 4, Supplementary Figure S5). The LAMARC results also indicated asymmetric migration between basins and sub-basins. The western Mediterranean sub-basins seemed to receive immigrants from the Atlantic and eastern Mediterranean basins; whereas migration in the opposite direction was negligible. Confidence intervals of the highest probability density (HPD) at 95% included the maximum value accepted by the program priors, indicating some limitations in the analysis. This was the case in the migration analysis from eastern to western Mediterranean, and in the demographic analysis of the eastern Mediterranean sub-basin and the Atlantic basin.

**Figure 7:**
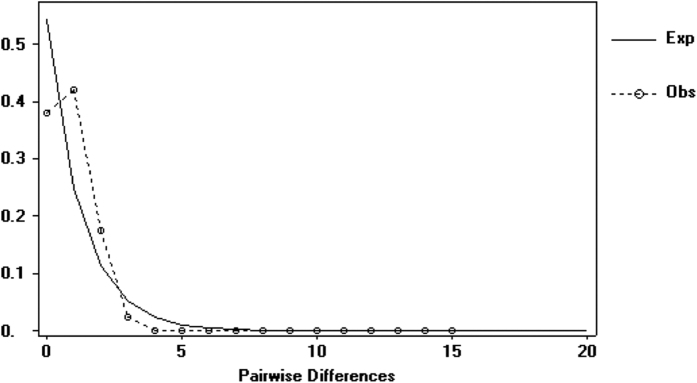
Mismatch distribution of *E. sepositus* for all samples. Observed (Obs) and expected (Exp) values of the distribution are represented as dashed and solid lines, respectively.

The unimodal mismatch distribution for the whole dataset revealed a peak close to the y-axis, which indicates recent expansion (Fig. 7). Bayesian analysis using LAMARC with the COI data supported the expansion model in two of the three basins. Estimations of demographic expansion based on different substitution rates for starfish dated this event between 9,000 and 13,000 generations ago. Assuming one generation per year, this expansion happened between 7,000 and 11,000 years after the Last Glacial Maximum (LGM).

Other signals that could be interpreted as the result of a recent demographic expansion were also detected from microsatellite loci. The Wilcoxon test results showed a significant heterozygosis deficit related to the number of alleles in some populations; a common occurrence in populations in demographic expansion (Table 5). For the M-Ratio test, there were no values of M lower than the critical value, M_c_, suggesting no population decline at any of the sites.

## Discussion

In this study, by combining both nuclear and mitochondrial markers, we demonstrate that the intraspecific genetic structure of *E. sepositus* is characterised by historical processes of divergence and a recent demographic expansion. This is combined with the disruptive effect of contemporary oceanographic barriers between the Atlantic and Mediterranean basins, and between the western Mediterranean and eastern Mediterranean sub-basins. This species presents low values of genetic diversity, which is important for the future conservation of their populations.

The genetic diversity in *E. sepositus* was much lower than that observed in other echinoderms with planktotrophic larvae living in the same distribution area^24^,^25^,^51^,^55^,^58^,^59^. There are no previous studies of echinoderms with lecitothrophic larvae using the same genetic markers and geographical area available for comparison, but the haplotype diversity found for COI data was comparable to that of some other benthic groups, such as some colonial ascidians and molluscs, with very limited dispersal potential^9^,^60^. Low allelic richness has been explained in other marine species as a result of high levels of inbreeding and/or population decline^61^,^62^. Nevertheless, for *E. sepositus*, we did not detect signs of current inbreeding across the whole distribution range, although we did in a few subpopulations, and there was no evidence of recent bottlenecks. Therefore, an inbreeding depression or recent population reduction cannot explain the low diversity observed in *E. sepositus*. However, low genetic diversity characterises coastal areas of recent re-colonisation after the LGM, dated some 20,000 years ago across the northern hemisphere, where the signal of a strong founder effect still persists when populations have not yet reached equilibrium^17^,^31^,^48^,^63^,^64^; this might be the case of *E. sepositus*. A dramatic reduction of *E. sepositus* populations along the European coast during the LGM might explain its low values of genetic diversity.

**Figure 8:**
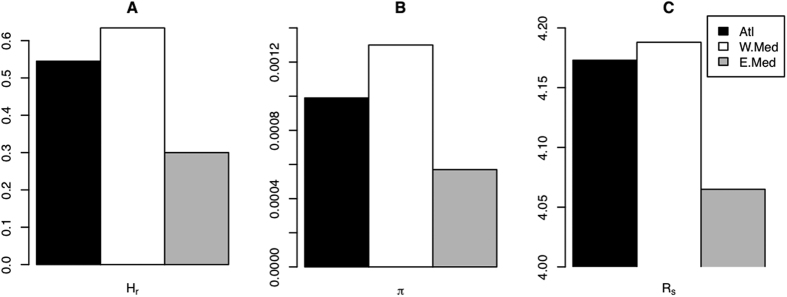
Bar plots representing diversity values from COI and microsatellites per basin. (**A**) Rarefied haplotype diversity, (**B**) nucleotide diversity, and (**C**) allelic richness. Black bars represent Atlantic populations (Atl), white bars western Mediterranean populations (W. Med), and grey bars eastern Mediterranean populations (E. Med).

Due to the differences in nature and coalescence time between mitochondrial and nuclear markers^65^, we need both types of markers to distinguish between past and contemporary processes involved in the phylogeographic patterns and genetic structure of the species. In *E. sepositus*, the COI data displayed genetic divergence in the structure of the population among the three oceanographic areas analysed: the Atlantic, western Mediterranean and eastern Mediterranean. This genetic divergence between basins and sub-basins was also supported by results from microsatellite loci. We detected sharp phylogeographic discontinuities, according to the AMOVA and genetic pairwise comparisons between populations, with the absence of some ancestral haplotypes in the Atlantic basin and the easternmost section of the Mediterranean, and the additional presence of private alleles within the three marine regions. These genetic divergences typically evolve in response to long-term extrinsic barriers to gene flow^66^,^67^. Oceanographic barriers, such as the well-known Almeria-Oran Front between the Atlantic and Mediterranean basins, and the Siculo-Tunisian Strait between the western and eastern Mediterranean, limit gene flow between different hydrographic basins in *E. sepositus*, as extensively documented for other marine benthic invertebrates. However, although oceanographic breaks exist, they should be understood as transition breaks with a discontinuous barrier effect^68^, and populations at the edges between biogeographical areas may be influenced by oceanographic circulation between areas; as in the case of the populations at Taormina or Cartagena analysed in this study. The long-existing separation between Atlantic and Mediterranean haplotypes and a posterior recolonisation of the Mediterranean basin by Atlantic haplotypes would partially explain the higher haplotype diversity found in the western Mediterranean, as observed in other echinoderm species^51^. This hypothesis is supported by a clear unidirectional gene flow observed from the Atlantic to the western Mediterranean. A predominant superficial current across the Strait of Gibraltar from the Atlantic to the Mediterranean Sea^27^ promotes directional gene flow by dispersion of larvae. This oceanographic circulation pattern has probably favoured past secondary contact between basins after periods of divergence during the Pleistocene glaciations, partially homogenising the genetic structure between populations^51^,^69^. Additionally, a very recent demographic expansion in *E. sepositus*, estimated between 9,000 and 13,600 generations ago, after the LGM, has also shaped the phylogeographic patterns in this species; a pattern observed in a number of marine invertebrates across the North Atlantic^17^,^20^,^31^,^70^,^71^, even in other *Echinaster* species along the coast of Florida (North-western Atlantic)^72^, and the starfish genus *Acanthaster* from distance geographical areas of the Indian Ocean^73^. This recent demographic expansion probably favoured geographical redistribution of the most frequent haplotypes and secondary contact, as discussed above. The greater genetic diversity for both nuclear and mitochondrial markers (Fig. 8), and the presence of the three ancestral haplotypes (H_1, H_2 and H_3) in the western Mediterranean basin, also points to this particular area as the central origin of the distribution of the species^64^,^74^. Nevertheless, other hypotheses cannot be ruled out, since a higher genetic diversity in the western Mediterranean might also be a consequence of the larger effective population size, or the influence of genetic inflow and genetic admixture from eastern Mediterranean and Atlantic sources.

However, our results based on the LAMARC Bayesian approach suggest a stable migration for a long period^75^ and therefore some considerations must be taken into account. Results of the process of migration into the western Mediterranean could include an effect on the exponential expansion from this basin to other basins. Additionally, in order to translate the N_f_ values into absolute population sizes, we note that N_f_ only counts mature females, and we have no information on the age of maturity of the species. Finally, results for the estimated number of individuals were within a range, and different markers in MCMC analysis already reported variation within a species^76^. Nevertheless, the larger population size could indicate better environmental conditions for this species along the western Mediterranean area, reinforcing the hypothesis of the origin of expansion in this basin.

The genetic structure of the populations in *E. sepositus* did not follow a pattern of IBD when the disruptive effect of marine fronts between basins was eliminated^57^. Aquarium experiments in *E. sepositus* showed that both gametes and larvae immediately sink to the bottom of the experimental tanks^77^ after induced spawning, although floating eggs are also reported elsewhere for *E. sepositus*^42^,^43^. Hence, although some experimental data and literature seem contradictory for *E. sepositus*, the release of both floating and non-floating eggs has been observed in other *Echinaster* species during the same reproductive event^78^. The existence of gametes and larvae with different behaviour and/or dispersal potential (floating versus non-floating) in *E. sepositus* might explain the low differentiation between populations due to high connectivity promoted by floating and long-dispersal gametes and larvae. Additionally, the absence of IBD could be related to the stochastic nature of larval connectivity in nearshore species due to intermittent and heterogeneous processes of marine circulation, which vary over time, habitat availability and other biological features, such as competition and predatory pressure^13^. These complex systems of physical and biological interactions can generate large variability in the recruitment process, complicating connectivity networks over time^13^. Hence, further studies of larval behaviour and the ultrastructure in *E. sepositus* are needed to understand their relevance to the connectivity of populations and the potential evolutionary significance of divergent strategies of dispersal.

Interestingly, two Mediterranean populations, Livorno and Cartagena, displayed sharp differences in genetic structure based on microsatellites, and appeared as separate clusters. Those differences were less noticeable in mtDNA sequences, which may indicate that the divergence of these two populations is a relatively recent process. These two particular populations are located close to large marine harbours, and coastal areas affected by industry. In the case of Livorno, just 11 km away from our sampling site there is a famous white beach produced by the industrial carbonate discharges of Rosignano Solvay. The load of heavy metals around the Livorno harbour has been demonstrated to impact genetic diversity of other marine species^79^,^80^. The population at Cartagena is located just 15 km away from an iron mine that deposited tones of heavy metal-rich sediments over several decades^81^. Although our experimental design does not allow us to assess the effects of pollutants on the genetic structure of *Echinaster*, those effects may be related in some way with the low diversity and divergent structure found at these two particular sites. The genetic structure of populations in polluted areas may be due to the existence of selective sweeps^82^ or changes in the reproductive biology of the population with effects on the population structure^83^. Further studies considering both neutral and non-neutral markers such as single nucleotide polymorphisms (SNPs) could provide valuable information on potential adaptations to pollutants.

Population decline was not detected at any of the sites analysed, but the significant F_IS_ values, which resulted in Hardy-Weinberg disequilibrium, might be related with inbreeding in some populations. The general patterns of genetic structure in *E. sepositus*, together with the low values of genetic diversity detected and potential inbreeding, make this species highly vulnerable to overfishing and environmental perturbations at a small to medium geographical scale^71^,^84^. Uncontrolled and continued extraction of specimens from nature could dramatically reduce effective population size, increasing the risk of losing genetic diversity by genetic drift, which is more marked in small populations. This effect might be particularly important in *E. sepositus* due to the stochasticity of the recruitment process that does not ensure gene flow or recruitment of larvae from nearby sites, thereby limiting the recovery of populations.

The genetic data presented here for *E. sepositus* set the base for further studies to design management policies for sustainable exploitation of this iconic starfish. Any conservation strategy for this species should consider how genetic diversity is geographically distributed and the genetic divergence among the Atlantic, western Mediterranean and eastern Mediterranean basins.

## Methods

### Sampling

Tube feet samples from 332 specimens of *E. sepositus* were collected at 15 different locations: 9 in the western Mediterranean (Cabrera, Carboneras, Cartagena, Blanes, St. Feliu de Guíxols, Roses, Marseille, Livorno, Tabarka), four in the eastern Mediterranean (Taormina, Monastir, Rhodas, Rogoznica) and two in the Atlantic Ocean (Roscoff, Los Gigantes) (Fig. 2). The specimens were collected by scuba diving at depths of between 5 and 30 meters in most locations, and by trawling, at 100 metres, at the Mediterranean location of Blanes. Between 9 and 30 individuals per population were processed, depending on the abundance of the species at each location (See Table 1). We used a minimally invasive system for tissue collection, and animals were rapidly released back to the same place where they were collected. The tube feet were preserved in absolute ethanol and kept at −20 °C until processing.

### Genotyping and Sequencing

DNA was extracted from all tube feet samples using a REDExtract-N-Amp Tissue PCR Kit (Sigma-Aldrich, www.sigmaaldrich.com), following the protocol proposed by the manufacturer.

#### Microsatellites genotyping

We used eight specific microsatellite loci (mES_2, mES_4, mES_23, mES_24, mES_25, mES_29, mES_30, mES_38) already optimised for this species^85^. All the microsatellites were separately amplified by PCR with the exception of mES_4 and mES_30, which were amplified together in multiplex. Forward primers were labelled with a fluorescent dye^85^. All PCR amplifications were performed in 10 μl total volume, using 5 μl of the REDEXtract-N-Amp Tissue PCR Kit (Sigma-Aldrich), 5 pmol of each primer, 3.7 μl of ultrapure water, and 0.5 μl of DNA extraction. Thermal cycling was performed in a Bio-Rad S1000 dual thermal cycler (BioRad, www.bio-rad.com) with a first step of 95 °C for 60 sec, followed by 35 cycles of 95 °C for 20 sec, 50 °C for 20 sec and 72 °C for 2 minutes, and a final extension of 5 minutes. Amplification products were purified and analysed on an Applied Biosystems 3730xl DNA Analyzer (Applied Biosystems, www.appliedbiosystems.com) at the Scientific-Technical Services of the University of Barcelona. Allele length was estimated relative to the internal GENESCAN 400HD ROX size standard (Applied Biosystems) using Peak-Scanner software. Alleles were scored using the MsatAllel 1.0 package^86^ for R 3.1.

#### Sequencing of mitochondrial DNA

A fragment of the mitochondrial cytochrome *c* oxidase subunit I gene (COI) was amplified with the primers F210-CO1 (5′-GTAATGCCAATTATGATTGG-3′) and COA (5′-AGTATAAGCGTCTGGGTAGTC-3′)^87^. PCR reactions were performed in a total volume of 20 μl, using 8 μl of the REDEXtract-N-Amp Tissue PCR Kit (Sigma-Aldrich), 10 pmol of each primer, 8.4 μl of ultrapure water, and 1 μl of DNA extraction. Thermal cycling was performed in a dual thermal cycler as explained before at 96 °C for 90 sec; followed by 35 cycles of 96 °C for 20 sec, 48 °C for 80 sec and 72 °C for 90 sec, and a final extension of 5 minutes. Amplifications were visualised in agarose gels, and purification and sequencing of the PCR products were performed in a 3730xl DNA Sequencer at Macrogen services (www.macrogen.com) with the same primers used for amplification of the fragment. Sequences were edited and trimmed with MEGA v. 5.2 software^88^ and aligned with CLUSTALW. Sequences with singletons were re-amplified and sequenced again. The sequences obtained have been deposited in GenBank (accession numbers: KX792505-KX792527, www.genbank.com).

### Genetic diversity, structure and demography

#### The genetic descriptors

Haplotype number (Nh), nucleotide diversity (π) and haplotype diversity of COI, were calculated using the software DNASP v. 5.0^89^. Haplotype richness (Hr) was calculated after rarefaction to a size of 8, which was the size of the smallest population analysed, with CONTRIB v. 1.02^90^ in order to compare populations with different sample sizes. A network of COI sequences was reconstructed with Network v. 4.6 (Fluxus Technology, www.fluxus-engineering.com/sharenet.htm) using an infinite site model. Haplotype frequencies in each population were plotted in geographic coordinates using the packages Maps and Mapplots in R^91^,^92^.

Allele number per microsatellite locus, allele richness, observed heterozygosity (H_o_) and expected heterozygosity (H_e_) were calculated using the hierfstat package in R^93^. The fixation index F_IS_ was also calculated for each population and confidence intervals at the 0.05 level were tested after 100 bootstrapping resampling operations over loci with the same package^89^. The Hardy-Weinberg equilibrium per population and its significance were tested after 1,001 permutations in GenoDive^94^.

In order to infer major homogeneous genetic units (K) across the distribution range of the species, the software STRUCTURE v. 2.3^95^ was used for microsatellite data, and for the combination of microsatellite data and COI sequences. This software detects major genetic groupings based on a Bayesian clustering approach. The software was initially run with the whole dataset, using a non-admixture model, and with a K value ranging from 1 to 15, with the sampling site as a prior, due the low F_ST_ values obtained for this species (see Results section)^96^. However, since the admixture model makes the analysis more flexible, it was repeated with an admixture model using the location as a prior^97^. For sequences of COI, the second allele was treated as missing data, following the software manual for haploid loci. Twenty independent replicates of 10,000 MCMC each were run, and a 10,000 burn-in period applied following the optimised parameters for STRUCTURE software^98^. The most likely value of ‘real’ clusters was identified by comparing the rate of change in the likelihood of K (L(K)). The optimal K value per run was determined and graphically visualised using the ad hoc statistic ΔK^98^ with the software Structure Harvester^99^ following the Evanno method^98^. The results of the 20 runs from all K values were summarised and graphically illustrated using the pipeline of CLUMPAK^100^. In order to optimise the comparisons between the different STRUCTURE runs, we represented the results from the same number of K for each, using the best ∆K from the microsatellite analysis (Supplementary Figure S1).

Hierarchical analysis of the molecular variance (AMOVA) was performed with Arlequin v. 3.5^101^ in order to detect population groupings according to COI and microsatellite loci. Populations were *a priori* grouped within basins and sub-basins, which are considered different ecoregions^102^, to test the potential effect of the Strait of Gibraltar and the Siculo-Tunisian Strait. AMOVA was performed separately for COI and microsatellite loci, due to the different signal provided by the markers (see Results section). Populations were grouped as follows: Atlantic (Los Gigantes, Roscoff), eastern Mediterranean (Rogoznica, Rhodes, Monastir and Taormina) and western Mediterranean (Tabarka, Livorno, Marseille, Blanes, Roses, St. Feliu, Cartagena, Cabrera and Carboneras). Additionally, populations on the limit between the eastern and western Mediterranean (Taormina, Monastir and Tabarka) were interchanged between groups for the AMOVA, due to the difficulties in assigning these populations to a particular area. AMOVA based on microsatellites was run both including and excluding the populations at Livorno and Cartagena, because of the large genetic differentiation of these two populations due to unknown processes (see a full explanation in Results and Discussion).

Pairwise differences in genetic structure between locations were separately explored for COI and microsatellites by two different approaches; the Φ_ST_ for COI and F_ST_ for microsatellites, and the Jost’s D coefficients of dissimilarity^103^ for both COI and microsatellite loci. The Φ_ST_ and F_ST_ values were computed in Arlequin v. 3.5^101^, and the Jost’s D values in SPADE^104^ for the COI, and in DEMEtics R package^105^ for microsatellites. In all cases, p-values or confidence intervals were calculated after 1,000 permutations, and p-values were adjusted for multiple comparisons using Benjamini-Hochberg corrections^106^. In order to explore the potentially different divergence signals obtained from COI and microsatellites, we calculated the Pearson correlation between the Φ_ST_ and F_ST_ values. If mitochondrial and nuclear markers show similar trends in genetic divergence between populations, then the Φ_ST_ (from COI) and F_ST_ (from microsatellites) should be highly correlated. Additionally, the correlations between the Φ_ST_ and F_ST_ with the Jost’s D for COI and microsatellites, respectively, were also calculated to explore whether the two types of analysis yielded similar results. Dissimilarity genetic matrices based on Jost’s D coefficients were graphically represented using non-metric MDS for both kinds of markers with the Vegan R package^107^.

Genetic differentiation due to geographical distance (IBD) was calculated using the Mantel test procedure in the Vegan package^107^. We separately correlated the Φ_ST_ and F_ST_ dissimilarity matrixes for COI and microsatellites, respectively, and a matrix of geographical distances. Geographical distances were considered as the shortest linear distance in kilometres by sea. The IBD analysis was performed with all populations with a stratification method to consider the divergence associated with the “basin” factor: Mediterranean and Atlantic basins^57^. Analysis was performed including and excluding Livorno and Cartagena from the microsatellite dataset. The significance of the tests was evaluated by 1,000 permutations of individuals between populations and basins.

Migration between geographical areas, effective population size (expressed as *theta*), and demographic events, such as population growth or decline, were estimated with a Bayesian approach as implemented in LAMARC v. 2.1.9^108^ from COI data. We ran this analysis for the Atlantic basin and the two Mediterranean sub-basins separately. The best evolutionary model for our dataset was inferred with jModelTest 2^109^ and then implemented in LAMARC. An initial run was performed with default parameters as priors, followed by a series of runs adjusting those priors in order to find the optimal parameters for our data. Once optimal priors were obtained from the initial runs, they were implemented in a final run. The final run was based on three different 1,000,000 MCMC replicates each, and a burn-in period of 100,000. For each of the three replicates, four simultaneous heating searches were performed with relative temperatures of 1, 3, 7 and 11. Priors for the final analysis are presented in Supplementary Table S3. The Effective Sample Size (ESS) was visualised in Tracer v. 1.6^110^ to confirm a large enough number of independent simulations (over 250), and the Gelman and Rubin diagnosis with the *coda* R package^111^ was applied to test convergence of multiple MCMC runs^112^ (Supplementary Table S4). Migration rates (Mt) were expressed as the number of migrants per generation *Mt* = *m*/*μ*, where *m* is the migration rate per generation, *μ* is the substitution rate, and *θ* is the *theta* value. The *theta* value is defined as *θ* = *2N*_*f*_* μ*, where *N*_*f*_ is the effective population size for mitochondrial markers; we calculated *N*_*f*_ using a substitution rate of between 2.3% and 3.6% per million generations applied in other asteroids^17^,^32^,^33^,^54^,^113^. Hence, *theta* values (*θ*) and growth rates (*G*) are defined from the evolution of different *theta* values: *θ*_*t*_ = *θ*_*1*_^*−Gt*^. The high computational requirements of LAMARC in both time and memory, which cannot be parallelised in a super-computer cluster, did not allow us to analyse microsatellites with this software; thus, other methods had to be applied for microsatellite loci.

Demographic changes based on COI sequences were also inferred with a mismatch distribution^114^ in DnaSP. Sudden expansions are typically represented by a unimodal distribution in the mismatch distribution; while multimodal distributions represent populations at equilibrium. We used the formula τ = *2ut*^114^ to approximate the time in generations (*t*) of the demographic changes from the coalescent methods. We used the same lineage substitution rate as before, between 2.3% and 3.6% per million generations; due to a lack of information on the generation time of *E. sepositus*, we assumed 1 year per generation, as applied to different echinoderms^17^,^32^,^33^,^54^,^113^.

For microsatellite loci, recent population size changes were explored using two different approaches: the software Bottleneck v. 1.2^115^, and critical_M for the M-ratio test^116^. We ran Bottleneck in order to detect excess or deficiency of heterozygosity in our populations using an infinite allele model (IAM), and a stepwise mutation model (SMM). The significance of the tests was determined using the Wilcoxon signed rank test after 1,000 replicates. The heterozygosity excess method exploits the fact that allele diversity reduces faster than heterozygosity during a bottleneck, because rare alleles are lost rapidly and have little effect on heterozygosity, thus producing a transient excess of heterozygosity relative to that expected in a population of constant size^115^,^117^. Nevertheless, Bottleneck is more successful at detecting population expansions than population declines^118^. The M-ratio test, which is the ratio between the number of alleles and the range in allele size, is based on the fact that during a population decline, the microsatellite allele size range decreases more slowly than the number of alleles because only the less frequent alleles are lost due to genetic drift. Hence the M-ratio test calculates the M value, a ratio between the number of alleles and the range in allele size, and its significance, the critical M value (M_c_), was calculated after 10,000 simulations, and population decline is inferred when M_c_ is greater than the mean M value^116^.

## Additional Information

**How to cite this article**: Garcia-Cisneros, A. *et al*. Low genetic diversity and recent demographic expansion in the red starfish *Echinaster sepositus* (Retzius 1816). *Sci. Rep.* **6**, 33269; doi: 10.1038/srep33269 (2016).

## Supplementary Material

Supplementary figures

Supplementary tables

## Figures and Tables

**Table 1:** Populations of *E. sepositus*.

	Code	Coordinates	Cytochrome c oxidase I (COI)				Microsatellites (8 loci)							
			N_COI_	Nh	Hr	Π	N_m_	R_s_	Allele number	Private Alleles	Ho	He	F_IS_	H-W p-value
Western Mediterranean														
CABRERA, Balearic Islands, Spain	CAB	39.152487, 2.946320	20	5	0.679 (±0.080)	0.00136 (±0.00025)	24	4.271	60	1	0.661	0.775	0.146	0.001**
CARBONERAS, Andalusia, Spain	CAR	36.992822, −1.885877	23	6	0.700 (±0.061)	0.00154 (±0.00028)	24	4.039	57	0	0.657	0.734	0.105	0.001**
CARTAGENA, Murcia, Spain	PAL	37.628559, −0.695339	22	5	0.645 (±0.085)	0.00133 (±0.00024)	23	3.830	52	1	0.605	0.705	0.142	0.001**
BLANES, Catalonia, Spain	BLA	41.650626, 2.818985	20	6	0.742 (±0.074)	0.00166 (±0.00027)	8	3.822	38	0	0.531	0.739	0.281*	0.001**
St. FELIU, Catalonia, Spain	PSA	41.770913, 3.030400	30	4	0.598 (±0.082)	0.0014 (±0.00022)	26	4.082	57	0	0.493	0.735	0.328*	0.001**
ROSES, Catalonia, Spain	ROS	42.236866, 3.211675	22	3	0.567 (±0.051)	0.00093 (±0.00013)	24	3.959	56	2	0.561	0.733	0.234*	0.001**
MARSEILLE, Provence, France	MAR	43.276094, 5.335732	26	4	0.502 (±0.105)	0.0015 (±0.00027)	24	4.340	62	1	0.656	0.774	0.152	0.001**
LIVORNO, Tuscany, Italy	LIV	43.472328, 10.332079	23	2	0.522 (±0.033)	0.00075 (±0.00005)	25	2.994	36	0	0.507	0.607	0.165	0.002**
TABARKA, Jendouba, Tunisia	TAB	36.968067, 8.757856	9	4	0.694 (±0.147)	0.00169 (±0.00044)	10	3.991	43	0	0.587	0.734	0.200	0.003**
TAORMINA, Sicily, Italy	TAO	37.845190, 15.296036	24	3	0.507 (±0.093)	0.00095 (±0.00021)	24	4.093	56	2	0.605	0.746	0.188	0.001**
Eastern Mediterranean														
MONASTIR, Monastir, Tunisia	MON	35.787254, 10.838771	21	3	0.186 (±0.110)	0.0029 (±0.00018)	22	3.590	48	0	0.597	0.680	0.123	0.004**
RHODAS, Dodecanese, Greece	RHO	36.446022, 28.228543	25	3	0.227 (±0.106)	0.0036 (±0.00017)	24	3.866	57	1	0.667	0.721	0.075	0.030
ROGOZNICA, Dalmatia, Croatia	CRO	43.522663, 15.95418	20	4	0.195 (±0.115)	0.00046 (±0.00029)	24	4.169	58	1	0.699	0.773	0.096	0.008**
Atlantic Ocean														
LOS GIGANTES, Canary Islands, Spain	GIG	28.265344, −16.84775	18	5	0.667 (0.083)	0.00132 (±0.00025)	24	3.893	56	2	0.575	0.722	0.203*	0.001**
ROSCOFF, Brittany, France	RFF	48.753712, −3.972950	22	3	0.437 (±0.105)	0.00077 (±0.00021)	24	4.184	65	4	0.567	0.738	0.232*	0.001**
Mediterranean Sea			285	20	0.560 (±0.027)	0.00114 (±0.00008)		4.189	94	9	0.602	0.727	0.195*	0.001**
Western Mediterranean			195	15	0.634 (±0.025)	0.0013 (±0.00008)		4.188	86	7	0.586	0.728	0.219*	0.001**
Eastern Mediterranean			90	8	0.300 (±0.061)	0.00057 (±0.00013)		4.065	78	2	0.654	0.725	0.135	0.001**
Atlantic Ocean			40	6	0.545 (±0.069)	0.00099 (±0.00017)		4.173	75	6	0.571	0.730	0.229*	0.001**
Total			325	23	0.619 (±0.021)	0.00129 (±0.000)		4.224	100	—	0.598	0.727	0.169	0.001**

Population name, code, coordinates, number of COI sequences (N_COI_), number of haplotypes (Nh), rarefied haplotype diversity (Hr), nucleotide diversity (π), number of genotyped specimens with eight microsatellites (N_m_), microsatellite rarefied allelic richness (R_s_), microsatellite allele number, observed and expected heterozygosity (Ho and He), fixation index (F_IS_) (*p-value < 0.05) and p-values of the Hardy-Weinberg equilibrium (H-W p-values) for each location (**p-value < 0.01).

**Table 2:** Analysis of molecular variance (AMOVA) in *E. sepositus* for COI and microsatellite loci.

Source of variation	d.f.	SSD	% Variation	F-stat	F-value	p-value
***Mitochondrial DNA***						
Geographical regions Med vs Atl						
Within populations	309	105.531	55.57	Φ_st_	0.442	0.000
Among populations	13	13.402	5.18	Φ_sc_	0.085	0.000
Among groups	1	17.530	39.24	Φ_ct_	0.3924	0.009
Geographical regions W Med vs E Med vs Atl						
Within populations	309	105.531	70.69	Φ_st_	0.283	0.000
Among populations	12	4.93	1.82	Φ_sc_	0.025	0.054
Among groups	2	15.95	27.78	Φ_ct_	0.275	0.000
Taormina grouped with western Mediterranean						
Within populations	309	105.531	67.69	Φ_st_	0.323	0.000
Among populations	12	4.859	0.58	Φ_sc_	0.008	0.278
Among groups	2	24.562	31.73	Φ_ct_	0.317	0.000
***Microsatellite genotypes***						
Geographical regions Med vs Atl						
Within individuals	282	688.5	80.1	F_it_	0.199	
Within populations	269	934.537	16.19	F_is_	0.175	0.001
Among populations	11	59.383	1.5	F_sc_	0.015	0.001
Among groups	1	12.725	1.5	F_ct_	0.015	0.001
Geographical regions W Med vs E Med vs Atl						
Within individuals	282	689	80.6	F_it_	0.194	
Within populations	269	935.729	17.1	F_is_	0.175	0.001
Among populations	10	51.006	1.3	F_sc_	0.013	0.001
Among groups	2	21.201	1	F_ct_	0.010	0.003
Taormina grouped with western Mediterranean						
Within individuals	282	689	80.5	F_it_	0.195	
Within populations	269	935.724	17.1	F_is_	0.175	0.001
Among populations	10	47.171	1	F_sc_	0.010	0.001
Among groups	2	24.455	1.5	F_ct_	0.015	0.001

Populations were grouped as follows: Med (Mediterranean) vs Atl (Atlantic) groups; within the Mediterranean basins with W Med (western Mediterranean) vs E Med (eastern Mediterranean); and with Taormina grouped with the western Mediterranean. For AMOVA with microsatellite loci, the Livorno and Cartagena populations were not considered.

**Table 3:** Results of the standard and stratified Mantel tests.

Mantel tests	Standard		Stratified	
	r	p-value	r	p-value
COI	0.7552	0.0002*	0.7552	0.236
Microsatellites (all locations)	0.1202	0.1752	0.1202	0.263
Microsatellites (without LIV and CAR)	0.6179	0.0027*	0.6179	0.066

The coefficient of correlation (r) and p-values are presented for both types of analysis. *p-value < 0.05.

**Table 4:** Demographic parameters of *E. sepositus* based on COI data.

	Growth	θ value	N_f_
Atlantic Ocean	9,763 [3,035–15,000]	0.0092	194–304
Western Mediterranean	7,369 [200–14,191]	1.5740	33,274–52,081
Eastern Mediterranean	11,550 [5,997–15,000]	0.0114	241–377

Mean population growth (Growth) and 95% HPD interval in brackets, *theta* (θ) values estimated from LAMARC, and effective population size of females (N_f_) calculated from the *theta* values and substitution rates of 2.3–3.6%.

**Table 5:** Demographic analysis of *E. sepositus* based on microsatellite loci.

	Code	IAM	SMM	M	Mc
Mediterranean					
CABRERA, Balearic Islands, Spain	CAB	0.125	0.009*	0.76	0.65
CARBONERAS, Andalusia, Spain	CAR	0.037*	0.014*	0.83	0.65
CARTAGENA, Murcia, Spain	PAL	0.156	0.019*	0.73	0.60
BLANES, Catalonia, Spain	BLA	0.004*	0.002*	0.74	0.55
St. FELIU, Catalonia, Spain	PSA	0.004*	0.004*	0.84	0.66
ROSES, Catalonia, Spain	ROS	0.027*	0.004*	0.76	0.65
MARSEILLE, Provence, France	MAR	0.156	0.037*	0.74	0.65
LIVORNO, Tuscany, Italy	LIV	0.156	0.156	0.71	0.65
TABARKA, Jendouba, Tunisia	TAB	0.009*	0.006*	0.68	0.58
TAORMINA, Sicily, Italy	TAO	0.125	0.027*	0.71	0.65
MONASTIR, Monastir, Tunisia	MON	0.097	0.004*	0.79	0.64
RHODAS, Dodecanese, Greece	RHO	0.371	0.383	0.80	0.65
ROGOZNICA, Dalmatia, Croatia	CRO	0.273	0.027*	0.77	0.65
Atlantic					
LOS GIGANTES, Canary Islands, Spain	GIG	0.014*	0.004*	0.78	0.65
ROSCOFF, Brittany, France	RFF	0.006*	0.004*	0.78	0.65
Atlantic		0.97	0.01*	0.79	0.69
Mediterranean Sea		0.99	0.002*	0.81	0.74
Western Mediterranean		0.99	0.004*	0.76	0.74
Eastern Mediterranean		0.97	0.01*	0.82	0.70

Results are based on the Infinite Allele Model (IAM) and Stepwise Mutation Model (SMM) using the Bottleneck software; and M values (M) and the critical value of M (*Mc*) from the M-ratio test.

*Significant when p < 0.05.
