# Supplementary material for: Low genetic diversity and recent demographic expansion in the red starfish Echinaster sepositus (Retzius 1816)
Source: Sci Rep. 2016 Sep 15;6:33269. doi: 10.1038/srep33269 (PMC5024105; doi:10.1038/srep33269)
Supplement: Supplementary figures [file srep33269-s1.doc]

**Low genetic diversity and recent demographic expansion in the red starfish *Echinaster sepositus* (Retzius 1816)**

**Alex Garcia-Cisneros^1,2*^, Creu Palacín^1^, Yousra Ben Khadra^3^, Rocío Pérez-Portela^2^**

**Supplementary material**

**Figures**

**Supplementary Figure S1.** Values of ΔK (Delta K) and L’(K) along the different K values obtained using STRUCTURE from two different datasets: A and C, from microsatellites; and B and D, from microsatellites and COI sequences combined.

**Supplementary Figure S2**. Comparison of STRUCTURE results from the combined dataset of COI and microsatellites applying different methods: A) an admixture method, and B) a non-admixture method. The analysis was always with location as a prior.

**Supplementary Figure S3.** Correlations between: (A) Φ_ST_ and Jost’s D for COI data and (B) F_ST_ and Jost’s D from microsatellite loci. Note the different scales of the graphs.

**Supplementary Figure S4**. Correlation between pairwise comparisons of populations using COI and microsatellite loci. A) Φ_ST_ of COI and F_ST_ of microsatellites, and B) Jost’s D from COI and microsatellites.

**Supplementary Figure S5.** Plots of MCMC values from LAMARC replicates for the Atlantic, western Mediterranean and eastern Mediterranean basins: A) *theta* values, B) Growth, and C) migration between basins and sub-basins.

**Supplementary Figure S6**. Values of Gelman and Rubin’s test that show whether the shrink factor converges between replicas or fluctuates.
