# Supplementary material for: Low genetic diversity and recent demographic expansion in the red starfish Echinaster sepositus (Retzius 1816)
Source: Sci Rep. 2016 Sep 15;6:33269. doi: 10.1038/srep33269 (PMC5024105; doi:10.1038/srep33269)
Supplement: Supplementary tables [file srep33269-s2.doc]

**Low genetic diversity and recent demographic expansion in the red starfish *Echinaster sepositus* (Retzius 1816)**

**Alex Garcia-Cisneros^1,2*^, Creu Palacín^1^, Yousra Ben Khadra^3^, Rocío Pérez-Portela^2^**

**Supplementary material**

Tables

|  | CABRERA | CARBONERAS | CARTAGENA | BLANES | ST. FELIU | ROSES | MARSEILLE | LIVORNO | TAORMINA | TABARKA | MONASTIR | RHODAS | ROGOZNICA | LOS GIGANTES | ROSCOFF |
| --- | --- | --- | --- | --- | --- | --- | --- | --- | --- | --- | --- | --- | --- | --- | --- |
| CABRERA | - | 0.013 | 0.11 | 0.091 | 0.297 | 0.011 | 0.34 | 0.02 | 0.199 | 0.286 | **0.526** | **0.448** | **0.525** | 0.198 | 0.16 |
| CARBONERAS | 0.000 | - | -0.037 | -0.066 | 0.059 | -0.014 | 0.103 | -0.001 | 0.031 | 0.021 | **0.299** | **0.24** | **0.298** | **0.324** | **0.351** |
| CARTAGENA | 0.048 | -0.025 | - | -0.053 | 0 | 0.003 | 0.016 | 0.013 | -0.028 | -0.03 | 0.162 | 0.116 | 0.16 | **0.515** | **0.527** |
| BLANES | 0.030 | -0.033 | -0.031 | - | -0.009 | 0.032 | 0.048 | 0.048 | 0.012 | -0.054 | **0.247** | 0.199 | **0.245** | **0.418** | **0.479** |
| ST. FELIU | **0.144** | 0.028 | -0.005 | -0.008 | - | 0.143 | -0.019 | 0.15 | 0.001 | -0.072 | **0.112** | 0.088 | **0.111** | **0.641** | **0.707** |
| ROSES | 0.000 | -0.014 | -0.005 | 0.011 | 0.089 | - | 0.15 | -0.041 | 0.042 | 0.141 | **0.279** | **0.215** | **0.278** | **0.414** | **0.341** |
| MARSEILLE | **0.192** | 0.061 | 0.007 | 0.026 | -0.021 | 0.112 | - | 0.153 | -0.009 | -0.041 | 0.051 | 0.032 | 0.05 | **0.741** | **0.777** |
| LIVORNO | 0.008 | -0.006 | 0.003 | 0.023 | 0.100 | -0.043 | 0.124 | - | 0.044 | 0.153 | **0.274** | 0.209 | **0.273** | **0.418** | **0.337** |
| TAORMINA | 0.118 | 0.015 | -0.027 | 0.002 | -0.005 | 0.029 | -0.015 | 0.034 | - | -0.011 | 0.082 | 0.046 | 0.081 | **0.634** | **0.622** |
| TABARKA | 0.105 | -0.009 | -0.034 | -0.044 | -0.060 | 0.070 | -0.043 | 0.088 | -0.020 | - | 0.123 | 0.098 | 0.121 | **0.611** | **0.706** |
| MONASTIR | **0.411** | **0.264** | **0.177** | **0.221** | **0.127** | **0.309** | 0.073 | **0.324** | 0.121 | 0.183 | - | 0.2 | 0 | **1** | **1** |
| RHODAS | **0.365** | **0.219** | 0.131 | **0.184** | 0.100 | **0.249** | 0.045 | **0.260** | 0.068 | 0.146 | -0.012 | - | 0.2 | **0.928** | **0.913** |
| ROGOZNICA | **0.402** | **0.256** | **0.170** | **0.213** | 0.121 | **0.301** | 0.068 | **0.316** | 0.115 | 0.172 | -0.024 | -0.014 | - | **1** | **1** |
| LOS GIGANTES | 0.088 | 0.124 | **0.207** | **0.140** | **0.265** | **0.207** | **0.344** | **0.227** | **0.309** | **0.208** | **0.583** | **0.555** | **0.574** | - | 0.03 |
| ROSCOFF | 0.109 | **0.206** | **0.307** | **0.251** | **0.390** | **0.250** | **0.466** | **0.264** | **0.408** | **0.380** | **0.686** | **0.653** | **0.680** | 0.033 | - |

**Supplementary Table S1**. Pairwise comparisons between populations of *E. sepositus* based on COI sequences. Φ_ST_ values (below the diagonal) and Jost’s D values (above the diagonal) are presented. Significant p-values (p-value < 0.01) are in bold.

|  | CABRERA | CARBONERAS | CARTAGENA | BLANES | ST. FELIU | ROSES | MARSEILLE | LIVORNO | TAORMINA | TABARKA | MONASTIR | RHODAS | ROGOZNICA | LOS GIGANTES | ROSCOFF |
| --- | --- | --- | --- | --- | --- | --- | --- | --- | --- | --- | --- | --- | --- | --- | --- |
| CABRERA | - | 0.002 | **0.06** | -0.006 | 0.004 | 0.003 | 0.007 | **0.125** | -0.001 | 0.018 | **0.036** | **0.021** | **0.02** | **0.024** | **0.014** |
| CARBONERAS | 0.013 | - | **0.054** | -0.003 | 0 | 0.002 | 0.007 | **0.133** | -0.004 | 0.006 | **0.027** | **0.013** | **0.019** | **0.039** | 0.011 |
| CARTAGENA | **0.154** | **0.139** | - | **0.056** | **0.056** | **0.062** | **0.025** | **0.087** | **0.042** | **0.084** | **0.069** | **0.082** | **0.055** | **0.056** | **0.05** |
| BLANES | 0.01 | 0.02 | **0.185** | - | 0 | -0.008 | 0.002 | **0.117** | -0.013 | 0.023 | **0.041** | **0.031** | 0.019 | **0.05** | 0.012 |
| ST. FELIU | 0.024 | 0.014 | **0.136** | 0.054 | - | -0.004 | 0.004 | **0.108** | 0.002 | 0.007 | **0.019** | 0.009 | **0.032** | **0.029** | **0.012** |
| ROSES | 0.033 | 0.035 | **0.171** | 0.026 | 0.014 | - | 0.01 | **0.102** | -0.007 | 0.017 | **0.028** | **0.015** | **0.029** | **0.041** | 0.006 |
| MARSEILLE | 0.035 | 0.042 | **0.081** | 0.053 | 0.019 | **0.058** | - | **0.072** | 0.004 | **0.023** | **0.037** | **0.025** | **0.016** | **0.039** | **0.015** |
| LIVORNO | **0.347** | **0.369** | **0.219** | **0.332** | **0.297** | **0.277** | **0.21** | - | **0.119** | **0.165** | **0.146** | **0.131** | **0.102** | **0.156** | **0.117** |
| TAORMINA | 0.013 | 0.004 | **0.122** | -0.01 | 0.024 | 0.001 | 0.036 | **0.42** | - | **0.022** | **0.022** | **0.02** | **0.023** | **0.034** | 0.007 |
| TABARKA | **0.067** | 0.034 | **0.229** | **0.097** | 0.058 | **0.084** | **0.102** | **0.33** | **0.098** | - | **0.029** | **0.031** | 0.016 | **0.069** | **0.039** |
| MONASTIR | **0.118** | **0.1** | **0.191** | **0.118** | **0.076** | **0.1** | **0.126** | **0.352** | **0.073** | **0.11** | - | **0.017** | **0.025** | **0.06** | **0.036** |
| RHODAS | **0.082** | **0.056** | **0.21** | **0.133** | **0.042** | **0.066** | **0.096** | **0.333** | **0.068** | **0.109** | **0.049** | - | **0.023** | **0.065** | **0.021** |
| ROGOZNICA | **0.078** | **0.07** | **0.166** | **0.094** | **0.115** | **0.098** | **0.076** | **0.282** | **0.084** | 0.05 | **0.064** | **0.074** | - | **0.073** | **0.038** |
| LOS GIGANTES | **0.1** | **0.145** | **0.156** | **0.201** | **0.114** | **0.162** | **0.151** | **0.411** | **0.134** | **0.246** | **0.204** | **0.224** | **0.261** | - | **0.028** |
| ROSCOFF | **0.064** | 0.046 | **0.134** | 0.089 | **0.06** | **0.045** | **0.075** | **0.33** | 0.034 | **0.136** | **0.11** | **0.087** | **0.124** | **0.111** | - |

**Supplementary Table S2**. Pairwise comparisons between populations of *E. sepositus* based on microsatellite loci. F_st_ values (below the diagonal) and Jost’s D values (above the diagonal) are presented. Significant p-values (p-value < 0.01) are in bold.

| LAMARC priors | Theta | Growth | Migration | | |
| --- | --- | --- | --- | --- | --- |
|  |  |  | Western Mediterranean | Eastern Mediterranean | Atlantic |
| Western Mediterranean | 1e10^-6^ – 10 | 200 – 15,000 | - | 3,000 – 10,000 | 2,000 – 9,000 |
| Eastern Mediterranean | 1e10^-4^ – 10 | 1,000 – 15,000 | 1 – 4,000 | - | Invalid |
| Atlantic | 1e10^-5^ – 10 | 50 – 15,000 | 1e10^-10^ – 2,500 | Invalid | - |

**Supplementary Table S3**. Priors selected for final analysis in LAMARC.

Values of theta were logarithmically transformed.

|  | Gelman and Rubin’s diagnosis | |  |
| --- | --- | --- | --- |
|  | Point est. | Upper C.I. | ESS |
| Ln. Data. Likelihood | 1.00 | 1.01 | 3,394 |
| Theta for western Mediterranean (WM) | 1.01 | 1.03 | 3,560 |
| Theta for eastern Mediterranean (EM) | 1.05 | 1.06 | 1,700 |
| Theta for Atlantic Ocean (AT) | 1.01 | 1.03 | 2,135 |
| Migration rate into WM from EM | 1.01 | 1.02 | 4,301 |
| Migration rate into WM from AT | 1.00 | 1.00 | 4,239 |
| Migration rate into EM from WM | 1.00 | 1.00 | 2,594 |
| Migration rate into AT from WM | 1.08 | 1.12 | 2,554 |
| Growth for WM | 1.00 | 1.00 | 36,81 |
| Growth for EM | 1.00 | 1.00 | 3,579 |
| Growth for AT | 1.00 | 1.01 | 2,763 |
| Multivariate psrf = 1.02 | | |  |

**Supplementary Table S4.** Gelman and Rubin’s test and effective sample size (ESS) analysis between replicas of the Bayesian analysis performed using LAMARC.
